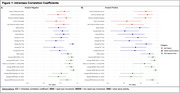# Reliability of longitudinal sleep monitoring in amyloid‐negative and amyloid‐positive older adults

**DOI:** 10.1002/alz70856_099179

**Published:** 2025-12-24

**Authors:** Taylor J Pedersen, Austin A. McCullough, Ruijin Lu, Cristina D Toedebusch, Ashley Hess, Rachel Richardson, Allyson Quigley, John C. Morris, David M. Holtzman, Brian A. Gordon, Brendan P Lucey

**Affiliations:** ^1^ Washington University School of Medicine, Saint Louis, MO, USA; ^2^ Washington University School of Medicine, St. Louis, MO, USA; ^3^ The Charles F. and Joanne Knight Alzheimer Disease Research Center, St. Louis, MO, USA; ^4^ Washington University School of Medicine in St. Louis, St. Louis, MO, USA; ^5^ Knight Alzheimer Disease Research Center, St. Louis, MO, USA

## Abstract

**Background:**

Sleep disturbances have been associated with both Alzheimer disease (AD) pathology and cognitive symptoms but there are few studies with longitudinal measures of sleep, AD biomarkers, and cognitive performance. To design and adequately power future aging and AD research, it is crucial to understand how sleep measures change in older adults with and without AD pathology.

**Method:**

Sleep was assessed longitudinally (∼3.5 years between visits) in amyloid‐negative and amyloid‐positive older adults. Data was collected using self‐reported sleep logs and questionnaires and with multiple nights of at‐home monitoring with a single‐channel electroencephalography (EEG) device (Sleep Profiler, Advanced Brain Monitoring, Carlsbad, CA). Amyloid PET imaging and standardized cognitive assessments were also performed longitudinally. Test‐retest reliability of the sleep metrics was measured using intraclass correlation coefficients (ICC) and a one‐sample power analysis was used to estimate sample sizes for longitudinal observational studies. As follow‐up, a factor analysis was conducted to identify the primary components of sleep monitoring.

**Result:**

In both amyloid‐negative and amyloid‐positive older adults, measures of spectral power derived from EEG had the highest mean test‐retest reliability followed by EEG‐derived sleep staging and duration then self‐reported sleep measures. Figure 1 illustrates the ICC values for each sleep measure across amyloid groups, reflecting their respective reliabilities. Sample sizes varied by sleep metric based on the average rate of change per year. Measures of spectral power showed the smallest samples size needed to detect longitudinal changes. A factor analysis of the collected measures identified five unique sleep factors.

**Conclusion:**

Objective EEG measures showed the greatest reliability over time in older adults with and without amyloid positivity. These findings provide valuable insights for the design of future longitudinal studies exploring the relationship between sleep and AD.